# Plastic Flooring as a Bedding Alternative: A Welfare Trade‐Off for Broiler Chickens

**DOI:** 10.1111/asj.70133

**Published:** 2025-11-30

**Authors:** Bruna Barreto Przybulinski, Maria Fernanda de Castro Burbarelli, Felipe Cardoso Serpa, Irenilza de Alencar Naas, Jean Kaique Valentim, Claudia Marie Komiyama, Fabiana Ribeiro Caldara, Rodrigo Garófallo Garcia

**Affiliations:** ^1^ Faculty of Agricultural Sciences Universidade Federal da Grande Dourados Dourados Brazil; ^2^ Posgtaduate program in Production Engineering Universidade Paulista São Paulo Brazil; ^3^ Department of Animal Production, Institute of Animal Science Universidade Federal Rural do Rio de Janeiro Rio de Janeiro Brazil

**Keywords:** antimicrobial, degeneration, dyschondroplasia, lesions, temperature

## Abstract

This study evaluated the behavior and incidence of locomotor disorders in broiler chickens raised on plastic flooring (PF) with and without a nanotechnological antimicrobial additive compared with a conventional system using wood shavings. A total of 1500 male Ross 408 lineage broilers were used, arranged in a completely randomized design with five treatments, wood shavings (WS), PF, PF + WS, plastic flooring with additive (PFA), and PFA + WS, and six replicates. Behavioral and thermographic assessments, body surface, foot and bed temperature, and locomotor health were performed on different days. No effect of bedding type on body temperature was observed; however, birds in the PFA group exhibited lower foot temperatures on Day 8. At 40 days, birds raised exclusively on PF or PFA exhibited longer sitting times, poorer locomotor scores, and a higher incidence of lesions. However, for broilers raised for up to 42 days, the exclusive use of PF resulted in locomotor impairment and aggravated joint lesions, suggesting the need for associations with WS to mitigate such effects.

## 1 Introduction

Chicken feet are a significant product for specific import markets, such as China (Santana et al. 2021), where they are highly valued due to their high collagen content. However, the high incidence of pododermatitis, a condition that compromises the integrity of the plantar skin (Teixeira et al. 2019), results in significant product devaluation, negatively affecting exporters' competitiveness and commercial profit margins.

Furthermore, genetic improvement aimed at rapid weight gain and greater feed efficiency has contributed to increased locomotor problems, such as tibial dyschondroplasia and other joint deformities. Accelerated growth exerts excessive pressure on birds' skeletal and muscular systems, which are often unable to keep pace with bodily development. As a result, their mobility is compromised, negatively impacting productivity and meat quality and raising concerns related to management and production sustainability (Weeks et al. 2000; Fidan et al. 2020). Since birds affected by locomotor disorders experience consequences such as pain, hunger, thirst, and an inability to express natural behaviors, they become more susceptible to other diseases and injuries, including those in the hocks and breast, severely compromising their physical and psychological welfare.

The quality of bedding material is crucial for broiler welfare, especially since birds spend most of their lives in direct contact with this surface, particularly after three weeks of age, when they spend more time lying down (Boussaada et al. 2022). The high moisture content of conventional plant‐based bedding frequently exacerbates contact dermatitis in birds. To mitigate this problem, alternatives such as slatted floors have been investigated. However, these findings raise other concerns regarding animal welfare (Garcês et al. 2013; Chuppava et al. 2018; Çavdarci et al. 2022), making it essential to carefully evaluate and elucidate aspects such as effects on thermoregulation, mainly during the initial rearing phases, behavior, and the incidence of locomotor problems. Plastic floorings (PFs) offer advantages in terms of durability and cost‐effectiveness, as they do not require frequent replacement and are easy to install and clean.

Their use in strategic areas, such as underdrinkers, has been shown to benefit litter quality, particularly during the first weeks of life, reducing the incidence of pododermatitis (Farghly et al. 2018; Sonnabend et al. 2022). Another challenge with conventional bedding is microbiological contamination, which can exacerbate contact dermatitis, hock lesions, foot problems, breast lesions, and other sanitary issues (Shepherd and Fairchild 2010). In this context, PF has emerged as a promising alternative, particularly when combined with nanotechnologies to incorporate antimicrobial agents.

The use of antimicrobial additives, such as zinc oxide, in PF enables greater pathogen control, minimizing biofilm formation and contributing to the reduction of diseases related to litter quality (Al‐Tayyar et al. 2020). Moreover, using PF as bedding material can improve bird cleanliness, resulting in cleaner feathers and lower feather cleanliness scores (Çavuşoğlu and Petek 2019), thus favoring their natural protection and health (Welfare Quality 2009).

In light of these considerations, this study evaluated behavior and the incidence of locomotor problems in broiler chickens raised on PF (with and without antimicrobial additives) as a substitute for conventional wood bedding.

## 2 Materials and Methods

The experiment followed the ARRIVE guidelines (ARRIVE Guidelines, Forthcoming), and all procedures adhered to current regulations. The Ethics Committee on Animal Experimentation of the Federal University of Grande Dourados (CEUA‐01/2021) approved the protocol.

### 2.1 Housing and Experimental Design

This study was conducted in an experimental poultry house situated in the city of Dourados, Mato Grosso do Sul, Brazil. The region is situated at latitude 22°13′18“ S, longitude 54°48′23″ W, at an altitude of 430 m, with a humid tropical climate featuring a dry winter (Cwa) according to Koppen (1948). The experiment occurred during the Southern Hemisphere winter, with an average maximum temperature of 25.2°C, an average minimum temperature of 10.6°C, and an average relative humidity of 61.7% throughout the experimental period.

The poultry house contained pens measuring 4 m^2^ each and was equipped with nipple drinkers, tube feeders, curtains, and additional curtains to control the internal temperature. Each pen had a 250‐W infrared lamp for heating during the initial phase.

During the first 3 days of rearing, the feed was scattered on kraft paper placed on top of the bedding, making it easier for the chicks to access the food. For the first 7 days of rearing, a small chick feeder was used per pen, and fiberboard chick guards were placed around the chicks until day 10.

The lighting program consisted of 23 h of light and 1 h of darkness until day seven, gradually increasing to 6 h of darkness thereafter, as outlined in the Ross strain manual. Lighting was provided by 40 W bulbs, resulting in an intensity of 22 lx.

A total of 1500 1‐day‐old male Ross 408 broiler chicks with an initial average weight of 39.9 g were used. After weighing and uniformity adjustment, the birds were randomly assigned in a completely randomized design to five treatments based on the type of bedding material, with six replicates per treatment and 50 birds per replicate, and housed at a density of 12.3 birds/m^2^. The experimental diet, based on corn and soybean meal, was provided ad libitum according to the production phase, meeting the nutritional requirements proposed by Rostagno et al. (2017).

### 2.2 Treatments—Bedding Materials

**FIGURE 1:**
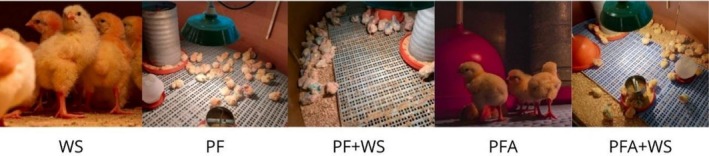
The experimental treatments included 100% wood shavings (WS), 100% plastic flooring (PF), 50% wood shavings and 50% plastic flooring (WS + PF), 100% plastic flooring with antimicrobial additive (PFA), and 50% wood shavings and 50% plastic flooring with antimicrobial additive (WS + PFA).

The bedding materials used were 100% wood shavings (WS), 100% PF, 50% PF + 50% WS (PF + WS), 100% plastic flooring with antimicrobial additive (PFA), and 50% PFA +50% WS (PFA + WS) (Figure 1). The bedding material used in the study consisted of new thermally treated and sieved Pinus WS. The noncommercial PF used was made of high‐density polyethylene (HDPE) with ultraviolet radiation protection, designed specifically for poultry pens. The flooring consisted of interlocking panels measuring 100 × 60 cm, with 12 × 12 mm perforations for the passage of excreta. The panels were suspended on profiles 20 cm above the concrete floor of the poultry house.

The additive incorporated into PF during manufacturing, utilizing nanotechnology, has antibacterial and antifungal properties. It was developed based on zinc oxide nanoparticles (MpZn_1300, TSNano, Florianópolis, Brazil). Nanotechnology involves creating microscopic particles embedded in plastic to enhance functionality and provide continuous antimicrobial activity. Zinc oxide nanoparticles possess large surface areas and high reactivity, effectively neutralizing bacteria, fungi, and some viruses.

Each pen measured 2.90 × 1.40 m (4.06 m^2^). The PF and PFA treatments covered the entire pen floor with PF. In the PF + WS and PFA + WS treatments, the PF covered a longitudinal strip of 2.90 × 0.70 m (2.03 m^2^), with WS covering the remaining sides, totaling 2.90 × 0.70 m (2.03 m^2^) of bedding area.

The WS in the WS, PF + WS, and PFA + WS treatments were stirred weekly without replacement of the material. No cleaning procedures were performed on the flooring throughout the experimental period.

### 2.3 Body and Bedding Temperatures During the Initial Phase

At 2, 8, and 15 days of age, body temperature was measured in three daily periods (6:00 a.m., 11:00 a.m., and 4:00 p.m.) using a clinical digital thermometer on two birds per replicate. The birds were gently restrained to avoid stress, and with their bodies held vertically, the thermometer was inserted into the cloaca. The temperature readings were taken immediately after the value stabilized.

The birds were subsequently carefully positioned to measure the superficial temperature of their feet using an infrared thermographic camera (TESTO, model 880, Testo SE & Co. KGaA, Lenzkirch, Germany). Images of both feet were simultaneously recorded at a 90° angle relative to their surface and an approximate distance of 50 cm. Following the literature for production animals, an emissivity value of 0.97 was adopted (McManus et al. 2022).

To determine the mean temperature, 20 random points on the feet of each bird were selected via Testo IRSoft software, following a methodology adapted from Garcia et al. (2019). At 8 and 15 days of age, infrared thermal images of the entire pen bedding surface were recorded at a distance of approximately 50 cm for all replicates during the same daily periods. Owing to the influence of heating by brooders, bedding images were not recorded at 2 days of age. To determine the mean surface temperature of the bedding, 30 random points were selected and evenly distributed across the image. An emissivity of 0.97 was used for WS, and 0.93 was used for PF, according to a methodology adapted from Nascimento et al. (2014).

Three digital thermohygrometers (Akso AK28, Akso Instruments Co. Ltd., Shenzhen, China) were installed along the poultry house at a height of 50 cm from the floor. Ambient temperature and relative humidity were recorded simultaneously each time thermographic images were captured to calibrate the camera measurements.

### 2.4 Behavior

For the behavioral assessment, 12 birds per treatment were individually identified with nontoxic paint across three replicates and evaluated at 3, 9, 16, 22, 29, and 40 days of age and 6:00 a.m., 11:00 a.m., and 4:00 p.m. During each period, three in situ observations were conducted at 30‐min intervals, recording the behavior exhibited by each bird at the exact moment of observation, according to an ethogram adapted from Lucena et al. (2020) and a methodology adapted from Dawson et al. (2021). The same trained evaluator performed all assessments throughout the study.

### 2.5 Feather Cleanliness Score

Feather cleanliness was evaluated following the Welfare Quality protocol (2009), which involves assessing the dirtiness of the breasts of two birds per replicate at 40 days of age. Scores were assigned as follows: (0) completely clean feathers, (1) slightly dirty, (2) moderately dirty in the central breast area, and (3) extensive dirt on the breast and wings.

### 2.6 Incidence of Locomotor Problems

For gait score, pododermatitis, hock lesion, angular deviation, and latency to lie analyses, 10 birds per replicate were identified and assessed at 20, 27, 34, and 41 days of age. Individual identification enables continuous evaluation over time and monitoring of lesion progression. The same evaluator conducted all scoring assessments.

Gait score evaluation was based on assigning scores to how birds walked within their housing pen, adopting a scale from 0 to 3: (0) normal walking, (1) walking with lameness or difficulty, (2) walking short distances and sitting down, requiring greater stimulus to move, and (3) unable to walk, as adapted from Webster et al. (2008).

Pododermatitis lesion scores were classified as follows: (0) no lesion, (1) small lesion on the plantar pad, (2) lesion covering the entire plantar pad, and (3) lesions on the toes and plantar pad, following Welfare Quality (2009).

Hock lesion scores ranged from 0 to 3: (0) no evidence of lesion, (1) minimal redness, (2) increased lesion area with necrosis onset, and (3) extensive burn and swelling in the hock area, according to Welfare Quality (2009).

Angular deviations of the legs relative to the central axis were measured via a protractor and ruler, which were used to evaluate the angle between the tibia and the third toe on both the right and left legs. Deformities were scored as (0) up to 10°, (1) 11°–20°, (2) 21°–30°, or (3) above 31°, adapted from Barbosa et al. (2022).

The latency to lie was assessed by placing 10 birds per round in two transparent containers filled with 3 cm of room‐temperature water. The time until the bird's first attempt to sit was recorded using a digital stopwatch, with a maximum time of 370 s, as reported by Almeida Paz et al. (2019).

At 42 days of age, the marked birds were fasted for 6 h and euthanized by cervical dislocation followed by bleeding, scalding at 58°C, de‐feathering, and eviscerating for macroscopic evaluation of tibial dyschondroplasia and femoral degeneration in both legs.

Tibial dyschondroplasia was assessed by observing growth plate cartilage thickening, which was scored as follows: (0) no thickening—no lesion, (1) thickening up to 3 mm—initial lesion, and (2) thickening over 3 mm—severe lesion (Almeida Paz et al. 2005). Femoral degeneration was scored from 0 to 2 by macroscopic examination of the femoral heads: (0) no lesion, (1) absence of cartilage and initial lesions, and (2) absence of cartilage and severe lesions (Almeida Paz et al. 2008).

The birds' backs were dissected and stored at −15°C for subsequent analysis of spondylolisthesis. A sagittal cut of the frozen vertebral column exposed the vertebrae, classified as lesion absent or present, as described by Paixão et al. (2007). The birds' feet were stored for later radiographic analysis.

Radiographs were compared with normal anatomy to detect bone alterations, changes in joint space, luxations, or fractures. Images were captured in dorsoplantar positioning via a JOB X‐ray portable high‐frequency X‐ray machine (Porta 120 HF) with 18 × 24 cm and 24 × 30 cm cassettes and a computerized radiography reader (CR) (Yan et al. 2005).

### 2.7 Statistical Analysis

The temperature data were assessed for residual normality using the Shapiro–Wilk test and for homogeneity of variance using Levene's test. Analysis of variance was performed via PROC MIXED, with flooring type as a fixed effect and the individual bird within treatment as a random effect (SAS 2015).

Nonnormal behavioral data were log‐transformed and analyzed with PROC GLIMMIX, which models the logarithm of the response variable as normally distributed. The time of evaluation was included as a covariate. Flooring type was included as a fixed effect, and individual bird was included as a random effect. Adjusted means were compared using least square means (LSM) with an inverse link function pairwise differences (PDIFF), and ILINK was applied to transform the mean from the model scale back to the data scale.

Locomotor scores and feather cleanliness data, which were nonnormally distributed, were analyzed via PROC GLIMMIX via generalized linear mixed models. The GAMMA distribution was used for most variables, whereas the BINOMIAL distribution was applied to spondylolisthesis and radiographic data.

Flooring type and bird age were treated as fixed effects with repeated measures, while the individual bird was considered a random effect. Analyses of tibial dyschondroplasia, femoral degeneration, and spondylolisthesis followed the same model. Regression coefficients and intercepts were obtained via the SOLUTION statement, and means were compared via lsmeans with inverse link adjustments (SAS 2015). Tukey's test was applied for multiple comparisons when significant differences were detected. The significance level was set at 5%.

## 3 Results

### 3.1 Body and Bedding Temperatures During the Initial Phase

The surface temperatures of the bedding materials and the body temperatures of the birds (Tables 1 and 2) did not differ among the different bedding types. No effect of bedding materials on the surface temperature of the feet was observed at 2 and 15 days of age. However, on Day 8 at 16:00 (4:00 p.m.), birds in the PFA group showed significantly lower foot temperatures compared to birds in the other groups (*p* = 0.007) (Table 3). No significant differences in foot or body surface temperature were observed at 2 and 15 days.

**TABLE 1:** Different bedding materials' surface temperatures (°C) at 8 and 15 days of age from infrared thermal images.

Days	Hours^a^	Bedding materials					SEM	*p*
		WS	PF	PF + WS	PFA	PFA + WS		
8	6 (14.8°C)	27.2	26.1	26.4	26.0	26.5	0.604	0.6092
	11 (5.5°C)	33.0	30.8	31.9	30.5	29.9	0.819	0.1298
	16 (19.2°C)	27.6	26.0	27.1	26.4	26.6	0.496	0.2151
15	6 (0.9°C)	15.9	15.3	16.1	14.8	15.5	0.648	0.6176
	11 (14.6°C)	24.4	23.1	23.7	24.3	23.7	0.830	0.8197
	16 (6.7°C)	23.7	21.6	22.4	23.9	23.0	0.828	0.2841

Abbreviations: PF, 100% plastic flooring; PFA, 100% plastic flooring with antimicrobial additives; PFA + WS, 50% wood shavings +50% plastic flooring with antimicrobial additives; SEM, standard error of the mean; WS, 100% wood shavings. Mean values in the same row followed by different lowercase letters differ significantly (*p*‐value < 0.05).

^a^ Ambient temperature at the time of evaluation is indicated beside the hours.

**TABLE 2:** Body temperature (°C) of broiler chicks raised on different bedding materials at 2, 8, and 15 days, obtained from thermographic images.

Days	Hours^a^	Bedding materials					SEM	*p*
		WS	PF	PF + WS	PFA	PFA + WS		
2	6 (13.1°C)	39.3	39.3	39.2	39.3	39.3	0.162	0.9469
	11 (23.0°C)	39.7	39.8	39.9	39.7	39.8	0.131	0.8807
	16 (16.9°C)	39.8	40.1	39.7	39.9	39.7	0.129	0.2142
8	6 (0.9°C)	39.5	39.7	39.8	40.0	39.6	0.166	0.3313
	11 (14.6°C)	41.0	41.0	40.9	40.9	40.9	0.126	0.8444
	16 (6.7°C)	40.3	40.1	40.2	40.4	40.4	0.131	0.3869
15	6 (0.9°C)	39.3	39.8	40.0	39.7	39.9	0.185	0.0851
	11 (14.6°C)	40.9	41.0	40.9	41.0	41.2	0.140	0.4643
	16 (6.7°C)	41.1	41.0	41.0	41.0	41.0	0.090	0.8611

Abbreviations: PF, 100% plastic flooring; PFA, 100% plastic flooring with antimicrobial additives; PFA + WS, 50% wood shavings +50% plastic flooring with antimicrobial additives; SEM, standard error of the mean; WS, 100% wood shavings. Mean values in the same row followed by different lowercase letters differ significantly (*p*‐value < 0.05).

^a^ Ambient temperature at the time of evaluation is indicated beside the hours.

**TABLE 3:** Surface temperature of the feet (°C) of broiler chicks raised on different bedding materials at 2, 8, and 15 days.

Days	Hours^a^	Bedding materials					SEM	*p*
		WS	PF	PF + WS	PFA	PFA + WS		
2	6 (13.1°C)	32.6	33.2	32.4	31.0	32.7	0.594	0.1269
	11 (23.0°C)	33.4	34.2	34.6	31.7	33.0	0.694	0.0539
	16 (16.9°C)	34.4	33.2	33.6	32.7	34.2	0.661	0.3501
8	6 (0.9°C)	32.9	32.6	31.7	34.2	32.5	0.748	0.2142
	11 (14.6°C)	35.7	36.0	35.0	34.1	35.6	0.548	0.1339
	16 (6.7°C)	35.3a	34.8ab	35.9a	33.9b	35.6a	0.334	0.0007
15	6 (0.9°C)	28.5	28.8	28.7	26.7	28.6	1.436	0.8242
	11 (14.6°C)	36.0	36.2	36.0	35.6	35.9	0.240	0.3919
	16 (6.7°C)	35.3	34.7	35.6	35.1	35.6	0.282	0.2145

Abbreviations: PF, 100% plastic flooring; PFA, 100% plastic flooring with antimicrobial additives; PFA + WS, 50% wood shavings +50% plastic flooring with antimicrobial additives; SEM, standard error of the mean; WS, 100% wood shavings. Mean values in the same row followed by different lowercase letters differ significantly (*p*‐value < 0.05).

^a^ Ambient temperature at the time of evaluation is indicated beside the hours.

### 3.2 Behavior

At 3 days of age, birds raised on PF with PFA presented more feather pecking behavior than those raised on the same type of flooring combined with WS (PFA + WS) (Table 4). Total or partial PF did not affect bird behavior at 9, 16, or 22 days of age (*p* > 0.05). At 29 days, birds raised on PF spent more time standing than those raised on wood bedding (WS) or PF + WS (*p* = 0.0496) (Table 5).

**TABLE 4:** Behavior frequency of broiler chicks raised on different bedding materials for 3 days.

Behavior	Bedding materials					SEM	*p*
	WS	PF	PF + WS	PFA	PFA + WS		
Standing	4.63	10.80	8.33	10.97	3.70	0.236	0.1734
Walking	3.40	2.47	2.47	4.15	2.78	0.203	0.8073
Sitting	75.62	70.68	66.67	61.34	72.53	0.086	0.2064
Eating	6.48	7.10	10.49	7.74	8.95	0.281	0.8476
Drinking	2.78	2.16	4.01	3.87	5.86	0.189	0.2649
Pecking bedding	2.78	3.40	3.40	3.59	3.09	0.260	0.8899
Feather exploring	1.85ab	2.16ab	2.77ab	5.13a	1.23b	0.129	0.0286
Comfort movements	2.47	1.23	0.93	2.86	1.23	0.346	0.2693
Scratching	0.00	0.00	0.00	0.00	0.00	—	1.0000
“Bathing” in bedding	0.00	0.00	0.00	0.00	0.00	0.001	1.0000
Aggressive pecking	0.00	0.00	0.00	0.00	0.00	—	1.0000
Nonaggressive pecking	0.00	0.00	0.93	0.34	0.62	0.001	1.0000

*Note:* The values are expressed as percentages of the total observations. The total percentages sum to 100% of the observations. Twelve birds per treatment were observed 3 times per period at 30‐min intervals for 36 evaluations. The percentages indicate the frequency of each behavior relative to the total observations. Mean values in the same row followed by different lowercase letters differ significantly (*p*‐value < 0.05).

Abbreviations: PF, 100% plastic flooring; PFA, 100% plastic flooring with antimicrobial additives; PFA + WS, 50% wood shavings +50% plastic flooring with antimicrobial additives; SEM, standard error of the mean; SEM, standard error of the mean; WS, 100% wood shavings.

**TABLE 5:** Behavior frequency of broilers raised on different bedding materials at 29 days of age.

Behavior	Bedding materials					SEM	*p*
	WS	PF	PF + WS	PFA	PFA + WS		
Standing	0.90b	3.18a	1.01b	1.54ab	1.68ab	0.273	0.0496
Walking	1.50	0.72	0.67	1.46	0.34	0.001	1.0000
Sitting	71.34	77.62	76.16	81.56	77.78	0.206	0.7498
Eating	8.22	6.84	7.58	6.73	7.27	0.227	0.9345
Drinking	6.87	4.73	3.77	3.05	3.47	0.201	0.1701
Pecking bedding	4.47	4.42	2.42	1.09	2.76	0.411	0.7617
Feather exploring	6.02	2.47	4.82	3.37	5.35	0.541	0.7072
Comfort movements	0.34	0.00	2.12	1.19	0.34	0.305	0.8514
Scratching	0.00	0.00	0.00	0.00	0.00	—	—
“Bathing” in bedding	0.34	—	1.45	—	1.01	0.459	0.8071
Aggressive pecking	0.00	0.00	0.00	0.00	0.00	—	—
Nonaggressive pecking	0.00	0.00	0.00	0.00	0.00	—	—

*Note:* The values are expressed as percentages of the total observations. The values are expressed as percentages of the total observations. The sum of the behaviors accounts for 100% of the compiled observations. Twelve birds per treatment were observed 3 times at 30‐min intervals for 36 evaluations. The percentage expresses the number of times the animal exhibited each behavior relative to the total number of observations. SEM: Standard error of the mean. Mean values in the same row followed by different lowercase letters differ significantly (*p* < 0.05).

Abbreviations: PF, 100% plastic flooring; PFA, 100% plastic flooring with antimicrobial additives; PFA + WS, 50% wood shavings +50% plastic flooring with antimicrobial additives; SEM, standard error of the mean; WS, 100% wood shavings.

At the end of the rearing period (40 days), broilers from the PF and PFA treatments showed a greater frequency of sitting behavior than birds from the whole or partial wood bedding treatments (WS, PF + WS, and PFA + WS) (*p* < 0.0001) (Table 6).

**TABLE 6:** The behavioral frequency of broilers raised on different bedding materials at 40 days of age.

Behavior	Bedding materials					SEM	*p*
	WS	PF	PF + WS	PFA	PFA + WS		
Standing	2.47	3.53	1.48	0.62	1.83	0.702	0.8968
Walking	1.44	0.00	0.74	0.62	0.74	0.169	0.4484
Sitting	84.05b	87.83a	84.07b	89.81a	82.85b	0.177	< 0.0001
Eating	4.94	2.29	3.70	3.09	4.47	0.273	0.3168
Drinking	3.60	0.00	2.96	1.85	3.95	0.344	0.0569
Pecking bedding	0.72	2.82	1.85	1.85	3.17	0.3116	0.0501
Feather exploring	1.54	3.53	4.82	1.54	2.99	0.357	0.5633
Comfort movements	1.24	0.00	0.37	0.62	0.00	0.589	0.6192
Scratching	0.00	0.00	0.00	0.00	0.00	—	—
“Bathing” in bedding	0.00	0.00	0.00	0.00	0.00	—	—
Aggressive pecking	0.00	0.00	0.00	0.00	0.00	—	—
Nonaggressive pecking	0.00	0.00	0.00	0.00	0.00	—	—

*Note:* The values are expressed as percentages of the total observations. The sum of behaviors accounts for 100% of the compiled observations. Twelve birds per treatment were observed 3 times at 30‐min intervals for 36 evaluations. The percentage expresses the number of times the animal exhibited each behavior compared to the total number of observations. Mean values in the same row followed by different lowercase letters differ significantly (*p* < 0.05).

Abbreviations: PF, 100% plastic flooring; PFA, 100% plastic flooring with antimicrobial additives; PF + WS, 50% plastic flooring +50% wood shavings; PFA + WS, 50% wood shavings +50% plastic flooring with antimicrobial additives; SEM, standard error of the mean; SEM, standard error of the mean; WS, 100% wood shavings.

### 3.3 Feather Cleanliness Score

**FIGURE 2:**
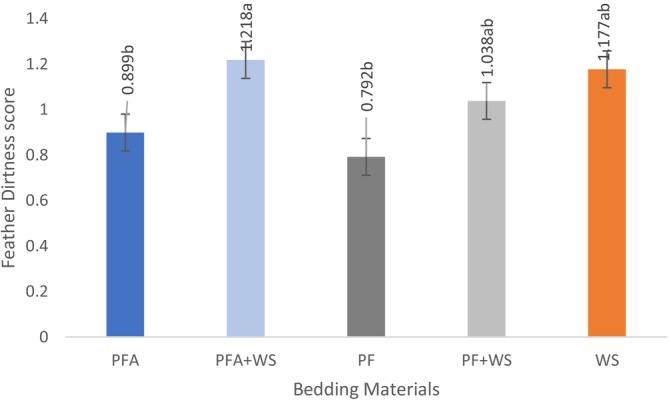
The feather cleanliness score of broilers raised on different bedding materials for 40 days. Effect of litter type on feather cleanliness score (least squares mean ± SE retransformed; higher values indicate greater abnormalities). Different letters (a, b) indicate statistically significant differences at *p* < 0.05. PF, 100% plastic flooring; PFA, 100% plastic flooring with antimicrobial additives; PF + WS, 50% plastic flooring +50% wood shavings; PFA + WS, 50% wood shavings +50% plastic flooring with antimicrobial additives; WS, 100% wood shavings.

At 40 days, birds housed exclusively on PF (PF and PFA) had significantly less feather soiling, as indicated by lower feather cleanliness scores, compared to those on the PFA + WS treatment (*p* = 0.0258) (Figure 2).

### 3.4 Incidence of Locomotor Problems

Antemortem evaluations of locomotor system health (gait score, latency to lie, pododermatitis, hock lesions, and foot angular deviation) revealed an interaction between bedding type and bird age over the rearing period (20, 27, 34, and 41 days). The regression equations are presented in the figures rather than described separately. These trends indicate a marked decline in locomotor health as birds age and gain weight. Additionally, the graphs show comparisons among bedding materials at each evaluated age.

At 27 and 34 days, bedding type did not influence gait score, indicating that the different materials did not affect locomotion differently at these ages. However, at 41 days, gait score analysis revealed significant differences among flooring types. Compared with those raised exclusively on PF (PF or PFA), birds raised on treatments containing WS (WS, PF + WS, or PFA + WS) had lower gait scores, indicating better walking ability (*p* < 0.001).

**FIGURE 3:**
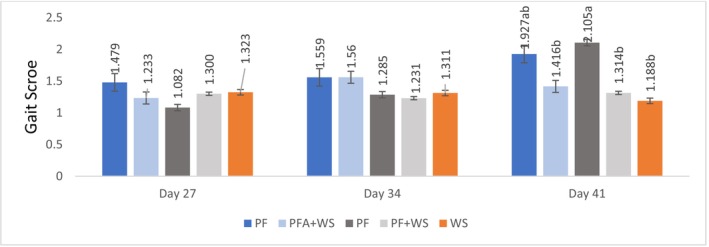
Gait scores of broilers raised on different bedding materials. Effect of litter type on gait score (least squares mean ± SE retransformed; higher values indicate greater abnormalities) on different evaluation days. Different letters (a, b) indicate statistically significant differences at *p* < 0.05. PF, 100% plastic flooring; PFA, 100% plastic flooring with antimicrobial additives; PF + WS, 50% plastic flooring +50% wood shavings; PFA + WS, 50% wood shavings +50% plastic flooring with antimicrobial additives; WS, 100% wood shavings. Interaction equations treatment*time, *p*‐value of the equation, and a: standard error of determination coefficients: MA: *y* = −0.003x^2^ + 0.278x − 4.506, *p* = 0.0046, *a* = 0.001 PP: *y* = 0.104x − 2.417, *p* < 0.0001, *a* = 0.011 PP + MA: *y* = 0.349x − 0.653, *p* > 0.0001, *a* = 0.010 PPA: *y* = 0.0676x − 1.284, *p* < 0.0001, *a* = 0.016 PPA + MA: *y* = 0.044x − 0.941, *p* < 0.0001, *a* = 0.010.

**FIGURE 4:**
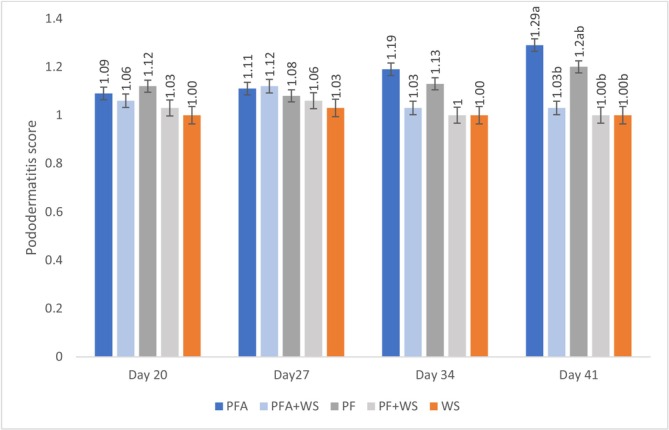
Pododermatitis scores of broilers raised on different bedding materials. Effect of litter type on pododermatitis (least squares mean ± SE retransformed; higher values indicate greater abnormalities) on different evaluation days. Different letters (a, b) indicate statistically significant differences at *p* = 0.0053. PF, 100% plastic flooring; PFA, 100% plastic flooring with antimicrobial additives; PF + WS, 50% plastic flooring +50% wood shavings; PFA + WS, 50% wood shavings +50% plastic flooring with antimicrobial additives; WS, 100% wood shavings. Interaction equations for treatment*time and standard error of determination coefficients: MA: Linear *p* = 1.000, Quadratic *p* = 0.1025. PP: *y* = 0.000522x^2^ − 0.02851x + 0.4768, *p* < 0.0425, *a* = 0.0003, *b* = 0.0191. PP + MA: Linear *p* = 0.6457, quadratic *p* = 0.0919. PPA: *y* = −0.00762x − 0.0788, *p* < 0.04321, *a* = 0.002. PPA + MA: *y* = 0.00035x^2^ + 0.01916x − 0.1775, *p* < 0.0193, *a* = 0.0002, *b* = 0.0170.

The gait scores decreased over time for all PF treatments, with the most pronounced increases observed in the PF and PFA groups (Figure 3). No effect of flooring type on pododermatitis lesions was observed up to 34 days of age. However, at 41 days, birds raised on PFA presented higher pododermatitis lesion scores than those raised on bedding containing wood bedding (WS, PF + WS, and PFA + WS) (Figure 4).

**FIGURE 5:**
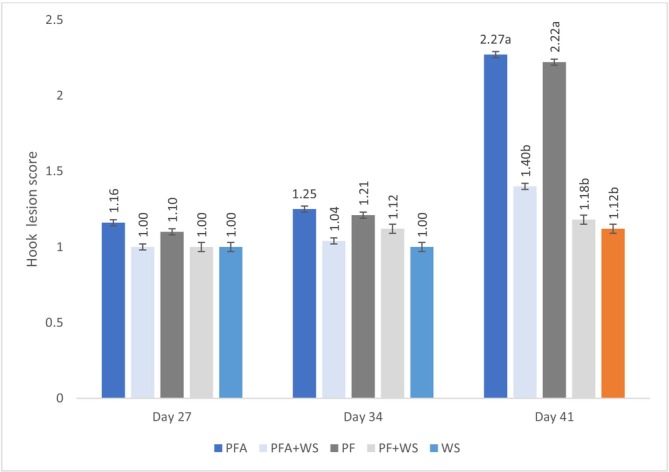
Hock lesion scores of broilers raised on different bedding materials. Effect of floor type on hock lesions (least squares mean ± SE retransformed; higher values indicate greater abnormalities) on different evaluation days. Different letters (a, b) indicate statistically significant differences at *p* < 0.0001. PF, 100% plastic flooring; PFA, 100% plastic flooring with antimicrobial additives; PF + WS, 50% plastic flooring +50% wood shavings; PFA + WS, 50% wood shavings +50% plastic flooring with antimicrobial additives; WS, 100% wood shavings. Data at 21 days were omitted from the graph because of the absence of hock lesions. Interaction equations for treatment*time and standard error of determination coefficients: MA: *y* = 0.009154x − 0.2695, *p* = 0.0006, *a* = 0.002 PP: *y* = 0.005288x^2^ − 0.3095x + 4.6016, *p* < 0.0001, *a* = 0.001, *b* = 0.059 PP + MA: *y* = 0.01148x − 0.2968, *p* < 0.0014, *a* = 0.003 PPA: *y* = 0.00536x^2^ − 0.3134x + 4.7612, *p* < 0.0001, *a* = 0.001, *b* = 0.06 PPA + MA: *y* = 0.002542x^2^ − 0.1486x + 2.1595, *p* = 0.0013, *a* = 0.001, *b* = 0.053.

No hock lesions were observed at 20 days; at 27 days, bedding type did not affect lesion scores. However, lesion progression was evident at 34 days in the groups raised exclusively on PF, becoming prominent at 41 days (Figure 5).

**FIGURE 6:**
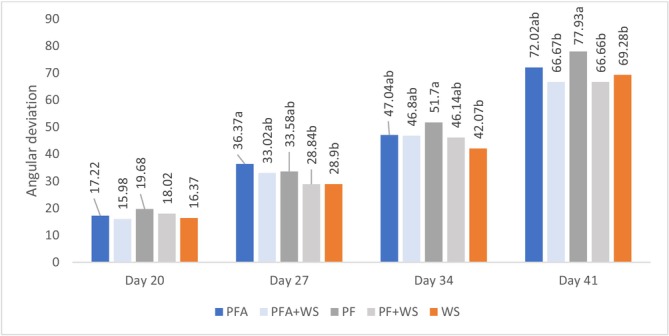
Angular deviation in broilers raised on different bedding materials. Effect of floor type on angular deviation (least squares mean ± SE retransformed; higher values indicate greater abnormalities) on different evaluation days. Different letters (a, b) indicate statistically significant differences at P ≤ 0.0001.PF, 100% plastic flooring; PFA, 100% plastic flooring with antimicrobial additives; PF + WS, 50% plastic flooring +50% wood shavings; PFA + WS, 50% wood shavings +50% plastic flooring with antimicrobial additives; WS, 100% wood shavings. Interaction equations for treatment*time and standard error of determination coefficients:MA: y = −2.4523x − 40.1200, *p* < 0.0001, *a* = 0.08 PP: *y* = 2.7269x − 41.8450, *p* < 0.0001, *a* = 0.07 PP + MA: *y* = 0.04902x^2^ − 0.6597x − 6.9605, *p* < 0.0054, *a* = 0.08 PPA: *y* = 0.02337x^2^ + 1.0591x − 16.7826, *p* = 0.0005, *a* = 0.07 PPA + MA: *y* = 2.3636x − 35.9691, *p* < 0.0001, *a* = 0.07.

**FIGURE 7:**
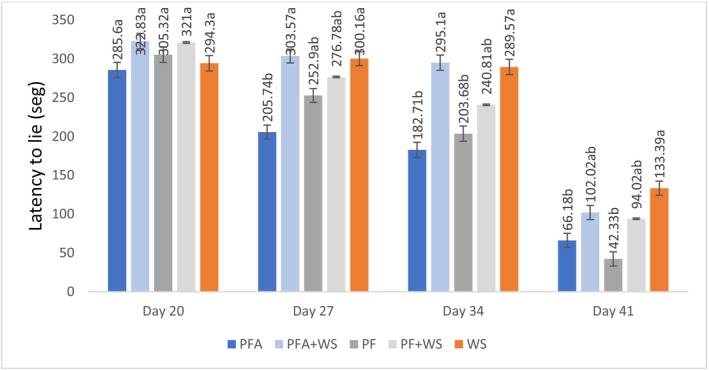
Latency to lie in broilers raised on different bedding materials. Effect of floor type on latency to lie (mean in seconds ± SE) on different evaluation days. Different letters (a, b) indicate statistically significant differences at *p* < 0.05. PF, 100% plastic flooring; PFA, 100% plastic flooring with antimicrobial additives; PF + WS, 50% plastic flooring +50% wood shavings; PFA + WS, 50% wood shavings +50% plastic flooring with antimicrobial additives; WS, 100% wood shavings. Interaction equations for treatment*time and standard error of determination coefficients: PPA: *y* = −9.330x + 471.09, *p* < 0.0001, *a* = 1.27 PPA + MA: *y* = −9.333x + 541.23, *p* < 0.0001, *a* = 0.911 PP: *y* = −11.477x + 551.79, *p* < 0.0001, *a* = 1.15 PP + MA: *y* = −9.716x + 528.10, *p* > 0.0001, *a* = 0.948 MA: y = −6.904x + 465.59, *p* > 0.0001, *a* = 0.951.

No differences in foot angular deviations were found at 20 days. However, from 34 days onward, significant deviations occurred in all the treatments, with greater increases in birds raised exclusively on PF (PF and PFA). At 20 days, the type of bedding did not affect the latency to lie. However, consistent with other locomotor health indicators, latency to lie decreased progressively from 34 days onward in all groups, with a more pronounced reduction in birds raised exclusively on PF (PF and PFA) (Figures 6 and 7).

**FIGURE 8:**
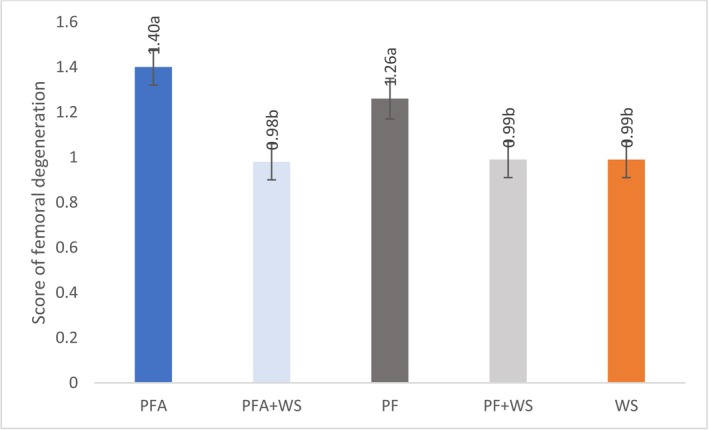
Femoral degeneration in 42‐day‐old broilers raised on different bedding materials. Effect of floor type on femoral degeneration (least squares mean ± SE retransformed; higher values indicate greater abnormalities). Different letters (a, b) indicate statistically significant differences at *p* < 0.0001. PF, 100% plastic flooring; PFA, 100% plastic flooring with antimicrobial additives; PF + WS, 50% plastic flooring +50% wood shavings; PFA + WS, 50% wood shavings +50% plastic flooring with antimicrobial additives; WS, 100% wood shavings.

**FIGURE 9:**
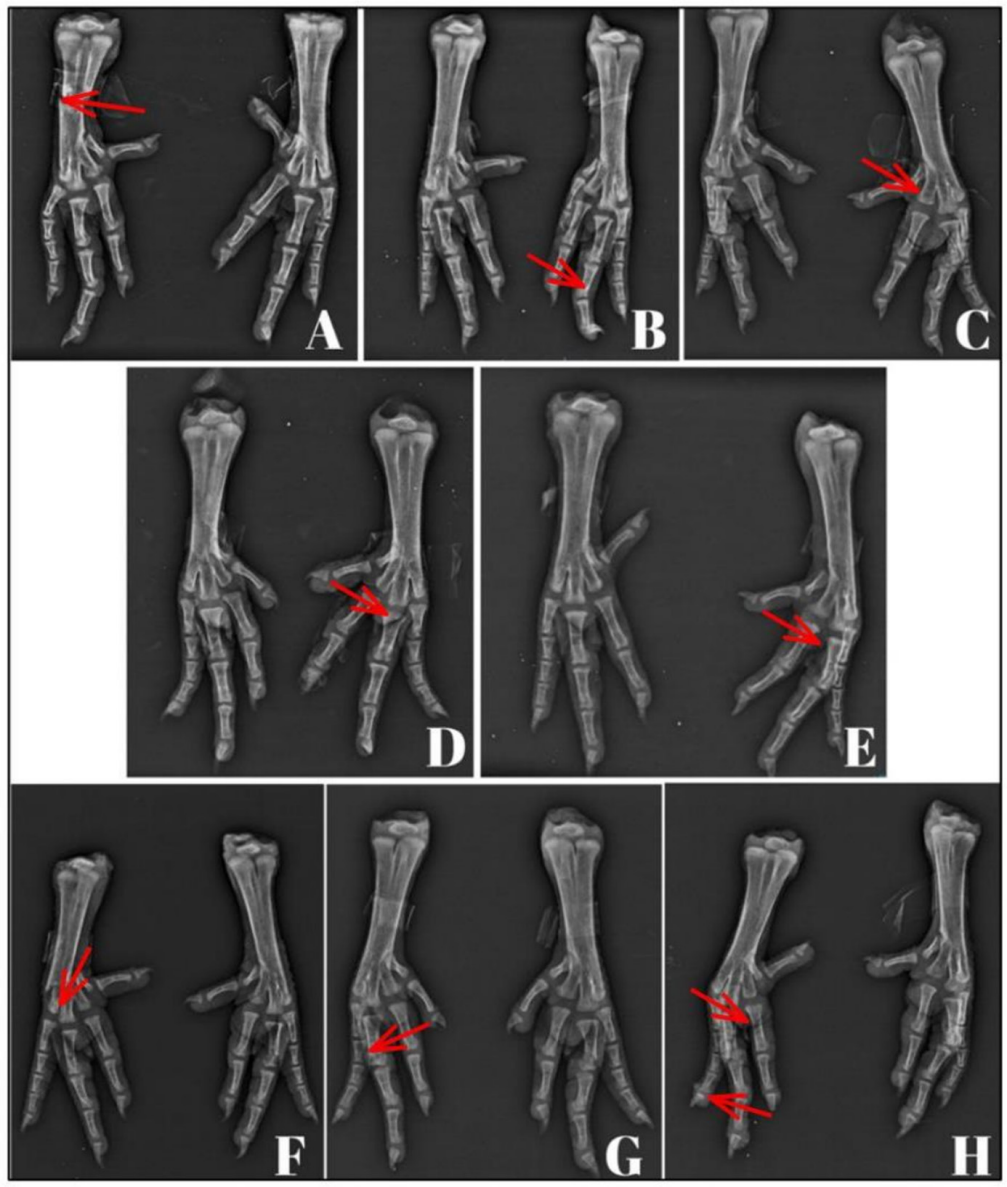
Different fractures were observed in the feet of 42‐day‐old broiler chickens raised on different litter materials. Panels A– show fractures in the WS treatment. Panels D and E represent fractures from the PF treatment. Panel F shows a fracture from the PF + WS treatment. Panel G shows a fracture from the PFA treatment, and Panel H shows a fracture from the PFA + WS treatment.

Compared with those raised on bedding with exclusive or partial wood bedding, birds raised exclusively on PF (PF or PFA) presented greater femoral degeneration scores (Figure 8). The bedding materials did not differ in terms of the incidence of tibial dyschondroplasia, spondylolisthesis, fractures, or foot lesions (Table 7). Simple and multiple fractures in the tarsometatarsus and phalanges were observed, with no significant differences noted among bedding materials (Figure 9).

**TABLE 7:** Tibial dyschondroplasia, spondylolisthesis, and fractures and dislocations in the feet of broiler chickens raised on different bedding materials at 42 days.

Variables	Bedding materials					*p*
	WS	PF	PF + WS	PFA	PFA + WS	
Tibial dyschondroplasia (right)	0	0	0	0	0	1.0000
Tibial dyschondroplasia (left)	0	0	0	0	0	1.0000
Spondylolisthesis	0.846	0.523	0.766	0.833	0.866	0.0625
Fractures and dislocations of the feet	0.952	0.974	0.983	0.941	0.983	0.7196

*Note:* The variables are represented as least squares means ± retransformed SE; higher values indicate greater abnormalities.

Abbreviations: PF, 100% plastic flooring; PFA, 100% plastic flooring with antimicrobial additives; PFA + WS, 50% wood shavings +50% plastic flooring with antimicrobial additives; WS, 100% wood shavings.

## 4 Discussion

The primary finding of this study is the pronounced, age‐dependent effect of flooring type on the welfare of broilers. While PF, both with and without the antimicrobial additive, appeared to be a viable alternative to WS for approximately the first 27 days, its exclusive use resulted in a significant and progressive decline in locomotor health as the birds aged and gained weight. After this point, birds on all‐plastic floors (PF and PFA treatments) began to exhibit significantly worse gait scores, a higher incidence and severity of hock lesions, greater femoral degeneration, and a more pronounced decrease in their latency to lie, indicating growing discomfort and impaired mobility.

This deterioration in locomotor health can be attributed primarily to the inherent physical characteristics of the PF. While the rigidity and smoothness of the PF demand greater effort from the birds to maintain balance, this can be attributed to the biomechanical challenges presented by the flooring, especially as the broilers' center of gravity shifts with age. This instability likely contributed to the higher incidence of angular leg deviations and, consequently, increased mechanical friction on the feet and hocks, leading to contact dermatitis. This physical discomfort is the most likely explanation for the observed increase in sitting behavior among birds on the PF and PFA treatments at 40 days. This inactivity creates a negative feedback loop, where birds sit for more extended periods, further exacerbating the lesions on their feet and hocks.

In stark contrast to these later‐stage issues, the PF systems did not negatively impact the birds during the initial rearing phase. For the first 15 days, the type of bedding had no significant effect on the birds' body or foot surface temperatures, which remained within the normal range for the species. Furthermore, no significant differences in key behaviors were observed among the treatments from Days 9 to 22. While weight gain certainly plays a role in locomotor health, it is plausible that differences in flooring materials contribute to the observed effects on gait and mobility, particularly as PF leads to greater mechanical stress.

During the initial phase of life, birds do not experience negative impacts from using PF, as their body temperatures remain within the normal range for the species (39.5°C–41.0°C). Additionally, foot surface temperatures were similar to those reported by Garcia et al. (2019), who found averages ranging from 32.2°C to 33.1°C. Jacob et al. (2016) noted variations between 35.9°C and 36°C, indicating that the temperatures in the birds in the present study were similar to those typically reported for broiler limbs up to 21 days of age. On the other hand, birds remained inactive for most of the initial period, spending more than 60% of their time sitting.

The bedding temperature likely contributed to this inactivity, as the temperatures recorded during many of the evaluated periods were below the ideal range for the environment and bedding in the initial production phase (24°C–30°C), as recommended by Aviagen (2018). Inactivity may be a strategy for birds to conserve body heat, minimizing heat exchange with the environment, as the bedding temperature is higher than the ambient temperature. During the experimental period, the outdoor temperatures near the houses ranged from 0.6°C to 23°C, which may have contributed to the low surface temperatures observed across the bedding materials in the initial phase.

Broilers exhibit innate behaviors such as foraging and dust bathing, and bedding material plays a crucial role in the frequency of these behaviors, which are closely linked to animal welfare (Jacobs et al. 2021). It was initially hypothesized that birds would exhibit exploratory behaviors, such as pecking, scratching, and dust bathing, more frequently in treatments containing WS; however, no changes in these behavioral patterns were observed from 9 days of age onward.

Conversely, feather pecking, a maintenance behavior involving rearranging feathers on the breast and wings with the beak (Chung et al. 2012), may be redirected in environments lacking suitable natural substrates for exploring bedding, leading birds to peck feathers instead (Johnsen and Vestergaard 1996; Riedstra and Groothuis 2004). This redirection may explain the increased frequency of feather pecking observed in birds from the PFA treatment group during the initial phase.

Immediately after hatching, broilers do not express their full behavioral repertoire, and their behavior is closely related to developmental stages (Costa et al. 2017). This growth‐related performance may explain the limited number of behaviors observed during this experiment, as birds primarily exhibited maintenance behaviors, such as eating and drinking, and spent long periods of inactivity without exploring the environment or interacting socially. Previous studies (Adler et al. 2020; Jacobs et al. 2021) also reported no significant behavioral differences during the first 2 weeks of life in broilers raised on partially perforated flooring, corroborating the results of this study.

The birds' behavior, particularly the increased time spent sitting in later stages, may be related to locomotor problem outcomes, where a pronounced effect of age and bedding material was observed. At 40 days, birds raised on PF or PFA spent more time sitting than those raised on WS, PF + WS, or PFA + WS. These findings suggest that PF may impair locomotion, resulting in reduced activity levels. A clear advantage of the all‐PF systems was observed in feather conditions. The results confirm that birds on PF and PFA floors at 40 days had significantly lower scores for the feather cleanliness score. This outcome was expected, as the perforated design allows excreta to fall through, thereby minimizing the birds' contact with wet litter, a primary cause of soiling in conventional systems (Li et al. 2017).

This finding has significant practical implications for producers, as improved feather cleanliness can lead to better carcass quality and potentially lower rates of downgrading at the processing plant. However, it is critical to weigh this advantage against the study's primary finding: the concurrent decline in locomotor health and welfare in older birds housed on these same hard surfaces. Until 27 days, bedding type did not influence locomotion parameters. However, the incidence of locomotor problems has increased over time, likely explained by rapid weight gain, which overloads the locomotor system and contributes to lesion development and worsening (Almeida Paz et al. 2010; Alves et al. 2016).

Increased body weight and muscle development, particularly in the breast, shift the broilers' center of gravity, potentially compromising their mobility and balance. PF, being smooth and less stable, demands greater effort from birds to maintain balance. During the experiment, a greater incidence of leg angular deviations (*valgus*) was observed in the PF and PFA treatment groups, accompanied by increased pododermatitis, hock lesions, and femoral degeneration, as well as decreased gait scores and angular deviations at 41 days.

Khan et al. (2023) described the biomechanics of broiler bones on WS and PF, reporting that birds raised on PF had a greater incidence of tibial dyschondroplasia, with smaller femur and tibia diameters and shorter lengths compared to those raised on other materials. These results reinforce the negative impact of PF on the skeletal system, suggesting that PF without WS increases locomotor problems in broilers.

Additionally, gait score assessment, used to quantify broiler locomotion capacity, has shown that changes in gait may be associated with discomfort, reduced physical activity, and compromised motivated behaviors, directly affecting bird welfare (Zhou et al. 2024). Birds on PF and PFA obtained the worst, consistent with previous studies reporting higher gait scores in birds raised on PF, likely due to reduced shock absorption and poor traction (Almeida et al. 2017).

Pododermatitis and hock lesions, classified as contact dermatitis, are related to bedding quality, as elevated ammonia, pH, and moisture levels aggravate these lesions (Kaukonen et al. 2016). Previous research (Adler et al. 2020; Sonnabend et al. 2022) reported higher pododermatitis scores in birds raised on WS beneath nipple drinkers than in those raised on partially perforated floors, likely due to increased moisture in the shavings. However, in the present study, increased sitting time and locomotor instability in the PF and PFA likely increased foot friction, contributing to lesion severity (Kaukonen et al. 2016).

The supposition that the nanotechnological antimicrobial additive would reduce cutaneous lesions, such as dermatitis, by controlling microbial load was not supported by our results. This finding strongly suggests that, in this context, the physical properties of the flooring, due to its hardness and lack of cushioning, were a more dominant factor in lesion development than the microbiological load. The increased sitting time and locomotor instability observed in birds on the PF and PFA floors likely increased the friction and pressure on their feet and hocks, contributing directly to the severity of contact dermatitis. Therefore, while zinc oxide nanoparticles are known to be effective against key bacteria implicated in pododermatitis (Olsen et al. 2018; Yusof et al. 2019), any potential antimicrobial benefits were insufficient to overcome the overriding negative impact of the mechanical stress induced by the floor itself.

Selecting the appropriate bedding material is essential to ensure broiler health, welfare, and performance, as it absorbs moisture, controls odor, promotes thermal comfort, maintains locomotor system integrity, and supports natural behaviors (Ghanima et al. 2020). Recent studies have investigated various plastic floor types for their ability to deter birds from excreting on them, promoting a drier and more hygienic environment with advantages such as durability, low operational costs, and ease of maintenance (El‐Maaty et al. 2023; May et al. 2024; Kaya et al. 2024). However, issues have been identified regarding the deficiency in behavioral *stimuli* and the adverse physiological outcomes, particularly concerning the locomotor system (Schomburg et al. 2023).

No behavioral or thermoregulatory changes were observed during the early rearing period. The exclusive use of PF resulted in a significant increase in locomotor disorders at the end of the production cycle. These lesions compromise locomotion, causing birds to sit for longer periods, which further exacerbates the problem. Such a scenario indicates welfare impairment, as rigid PF does not provide adequate cushioning, unlike WS, which offer a softer surface that reduces the impact on feet and joints.

Future research should address optimizing the concentration or type of antimicrobial agent. We also recommend further evaluation of the long‐term release kinetics and stability of the nanoparticles in a poultry house environment, as this was not the focus of the current study. Over time, an in‐depth microbiological analysis of the flooring surface would help detail the bacterial load and identify resistant strains, thereby reducing the need for antibiotics in animal production. The proposed research perspectives aim to explore ways to improve the impact of PF, focusing on reducing negative effects on animal welfare.

## 5 Conclusions

PF proved a viable alternative only during the initial rearing phase, showing effects comparable to those of WS on body temperature regulation, behavior, and locomotion. This method may be especially suitable for broilers slaughtered early (grillers) up to 28 days of age. However, in heavy broilers reared for 42 days, the exclusive use of PF had a negative impact on locomotor parameters, impairing gait, worsening joint deformities, and increasing the severity of pododermatitis, particularly on PF with the antimicrobial additive.

## Funding

This study was supported by the Coordination of Superior Level Staff Improvement for the scholarship of the first author (CAPES‐Finance Code 001), CAPES PDPG‐POSDOC 88887.799485/2022‐00, and the National Council for Scientific and Technological Development—CNPQ for scholarships PQ 304806/2022‐6, PQ 302254/2025‐0 and PQ 303934/2021‐2, and the Early Career Postdoctoral Fellowship (PDJ)—Call No. 2021/2023 (Process: 150188/2022‐6) and Call No. 32/2023 (Process: 177286/2023‐7).

## Conflicts of Interest

The authors declare no conflicts of interest.
